# Directed plant cell-wall accumulation of iron: embedding co-catalyst for efficient biomass conversion

**DOI:** 10.1186/s13068-016-0639-2

**Published:** 2016-10-21

**Authors:** Chien-Yuan Lin, Joseph E. Jakes, Bryon S. Donohoe, Peter N. Ciesielski, Haibing Yang, Sophie-Charlotte Gleber, Stefan Vogt, Shi-You Ding, Wendy A. Peer, Angus S. Murphy, Maureen C. McCann, Michael E. Himmel, Melvin P. Tucker, Hui Wei

**Affiliations:** 1 Biosciences Center, National Renewable Energy Laboratory, Golden, CO 80401 USA; 2 Forest Biopolymer Science and Engineering, USDA Forest Service, Forest Products Laboratory, Madison, WI 53726 USA; 3 Department of Biological Science, Purdue University, West Lafayette, IN 47907 USA; 4 X-ray Science Division, Advanced Photon Source, Argonne National Laboratory, Argonne, IL 60439 USA; 5 Department of Environmental Science and Technology, University of Maryland, College Park, MD 20742 USA; 6 Department of Plant Science and Landscape Architecture, University of Maryland, College Park, MD 20742 USA; 7 National Bioenergy Center, National Renewable Energy Laboratory, Golden, CO 80401 USA; 8 Department of Plant Biology, Michigan State University, East Lansing, MI 48824 USA

**Keywords:** Ferritin, Iron co-catalyst, Iron accumulation, Transgenic *Arabidopsis*, Biomass, High-throughput hot-water pretreatment, Saccharification, Sugar release, Perls’ Prussian blue staining, X-ray fluorescence microscopy

## Abstract

**Background:**

Plant lignocellulosic biomass is an abundant, renewable feedstock for the production of biobased fuels and chemicals. Previously, we showed that iron can act as a co-catalyst to improve the deconstruction of lignocellulosic biomass. However, directly adding iron catalysts into biomass prior to pretreatment is diffusion limited, and increases the cost of biorefinery operations. Recently, we developed a new strategy for expressing iron-storage protein ferritin intracellularly to accumulate iron as a catalyst for the downstream deconstruction of lignocellulosic biomass. In this study, we extend this approach by fusing the heterologous ferritin gene with a signal peptide for secretion into *Arabidopsis* cell walls (referred to here as FerEX).

**Results:**

The transgenic *Arabidopsis* plants. FerEX. accumulated iron under both normal and iron-fertilized growth conditions; under the latter (iron-fertilized) condition, FerEX transgenic plants showed an increase in plant height and dry weight by 12 and 18 %, respectively, compared with the empty vector control plants. The SDS- and native-PAGE separation of cell-wall protein extracts followed by Western blot analyses confirmed the extracellular expression of ferritin in FerEX plants. Meanwhile, Perls' Prussian blue staining and X-ray fluorescence microscopy (XFM) maps revealed iron depositions in both the secondary and compound middle lamellae cell-wall layers, as well as in some of the corner compound middle lamella in FerEX. Remarkably, their harvested biomasses showed enhanced pretreatability and digestibility, releasing, respectively, 21 % more glucose and 34 % more xylose than the empty vector control plants. These values are significantly higher than those of our recently obtained ferritin intracellularly expressed plants.

**Conclusions:**

This study demonstrated that extracellular expression of ferritin in *Arabidopsis* can produce plants with increased growth and iron accumulation, and reduced thermal and enzymatic recalcitrance. The results are attributed to the intimate colocation of the iron co-catalyst and the cellulose and hemicellulose within the plant cell-wall region, supporting the genetic modification strategy for incorporating conversion catalysts into energy crops prior to harvesting or processing at the biorefinery.

**Electronic supplementary material:**

The online version of this article (doi:10.1186/s13068-016-0639-2) contains supplementary material, which is available to authorized users.

## Background

Lignocellulosic plant residues are renewable, abundantly available materials that can be used to produce biofuels and biobased chemicals. However, there are still economic and technical challenges such as developing low-cost, mass production and transportation of biomass, and reduction of expenses associated with pretreatment and enzymatic hydrolysis of biomass to convert it into fermentable sugars [1]. Overcoming these crucial factors will constitute a major step toward widespread adoption of a renewable bioeconomy driven by industrial production of biomass-based fuels and chemicals. In this regard, feedstock genetic engineering is an integral part of green biofuels process, aiming to generate novel bioenergy crops to produce biomass with traits designed for easier downstream processing for the pretreatment and/or digestion steps [2].

So far, the vast majority of studies for genetically manipulating bioenergy crops have focused on modifying biosynthetic pathways in order to alter the content or composition of various biopolymers in plants. Foundational studies in this area demonstrated that lignin content [3] as well as monomeric composition [4] impacted the degradability of cell walls in forage material. More recent strategies for genetic manipulation of lignin focused on modifying its monomeric composition have produced materials that more deconstructable during pretreatment [5, 6], more susceptible to fungal decay [7], and variants that are more susceptible to enzymatic hydrolysis without any pretreatment [8]. Meanwhile, genetic manipulation of the carbohydrate components of the cell wall at the stage of biosynthesis are also attracting attention as promising routes to improve sugar yields in bioconversion processes [9, 10]. In contrast to these efforts to genetically modify the biopolymers of the cell wall, our present approach described in this study aims to incorporate deconstruction catalysts into the cell wall to enhance the yields of sugar release from conversion processes.

Previous studies have demonstrated that supplementation of iron ions as co-catalysts in dilute acid pretreatment of biomass can enhance the yields of sugars released in pretreatment and enzymatic digestion [11, 12]. The iron ions are found to promote degradation of multiple chemical bond types and compounds of the plant cell wall in pretreatment [13]. However, the approach of soaking or spraying the milled biomass with iron ion solution prior to pretreatment is not ideal due to labor and equipment costs as well as transport limitations for infiltrating dry plant tissue with iron ions. These infiltration problems arise from the strong binding of iron ions to biomass surfaces [14] and the presence of intracellular air-filled voids within cell-wall tissue. To more effectively overcome these process limitations, there is a need to explore novel approaches to engineer metal catalyst accumulation in plant feedstocks.

As an iron-storage protein, the following features of ferritin make it a strong candidate protein to be expressed in plants to produce more biomass with enhanced pretreatability and digestibility:

1. Ferritin has a high capacity for storing iron ions. The ferritin protein consists of 24 subunits and can bind up to 4300 iron atoms per ferritin molecule [15].
2. Heterologous ferritin proteins have been expressed intracellularly in rice [16–18], tobacco [19], and *Arabidopsis* [20] under the control of either endosperm-specific glutelin promoter or CaMV 35S promoter. The former promoter led to enhancements of iron and zinc accumulations in the seeds of transgenic rice [16–18], whereas the latter increased the iron concentrations in leaves of transgenic tobacco plants [19].
3. The intracellular overexpression of heterologous ferritin has been found to protect plants from photoinhibition and free iron toxicity, reduce oxidative stress [21–24], and improve the growth of transgenic plants [19, 25].

On the basis of the studies cited above that thoroughly investigated the effects of ferritin expression on iron accumulation and stress defense and growth in plants, our most recent study was the first attempt to engineer plants with intracellularly expressed heterologous ferritins (FerIN) to enhance plant biomass digestibility via *in planta* iron accumulation [26]. The objective of this study was to further advance the approach of *in planta* delivering metal co-catalyst into plant cell-wall region by expressing ferritin extracellularly (FerEX). We hypothesize that extracellular expression of heterologous ferritin allows iron to accumulate in proximity to the cell-wall matrix during plant growth, thereby promoting the intimate association of iron and biopolymers throughout the cell wall, which will eventually enhance the biomass post-harvest pretreatability. The literature reports support the feasibility of this approach as ferritin precursors with secretory signal peptide have been studied in insects and worms, by which ferritins are secreted out of the cells (see review [22]). In addition, native ferritin protein was found to be induced by dehydration in the extracellular matrix proteome of chickpea plant under drought stress [27], with a recent patent having been awarded for the possible role in enhancing plant drought resistance [28].

In this study, transgenic *Arabidopsis* plants (FerEX) were generated to extracellularly overexpress heterologous soybean ferritin protein, and can grow phenotypically normal (or better), and accumulate more iron ions during growth. The produced biomass had enhanced pretreatment and enzyme digestion yields to a larger extent than our previously generated *Arabidopsis* FerIN plants. The approach of *in planta* delivery of metal co-catalyst into the cell-wall matrix of plants distinguish itself from most other plant cell genetic engineering approaches that mainly focus on changing the composition of biopolymers or expressing cell-wall-degrading enzymes in plant cell wall for the enhancement of biomass digestibility.

## Results and discussion

### Ferritin transgenic *Arabidopsis* plants

Ten independent transformed T1 *Arabidopsis* FerEx plants that expressing soybean ferritin protein targeted extracellularly were generated. Total RNA was extracted from these ten transgenic lines and was reverse transcribed to cDNA. The prepared cDNA and the primers (listed in the “Methods” section) were used for the real-time RT PCR analysis, which detected the soybean ferritin transcripts in all ten transgenic lines.

### Shoot iron content and biomass yield of transgenic plants

Since iron accumulation is the main plant trait that is essential to the goal of this study, the initial measurement of iron content was conducted using the stems of these ten transformants at their T2 generation. Of these ten transformants, two transformants (FerEX-8a and -10g) showed the highest iron content, and were selected to further process to their T3 generation, for which their homozygosity was confirmed by segregation analysis.

**Fig. 1:**
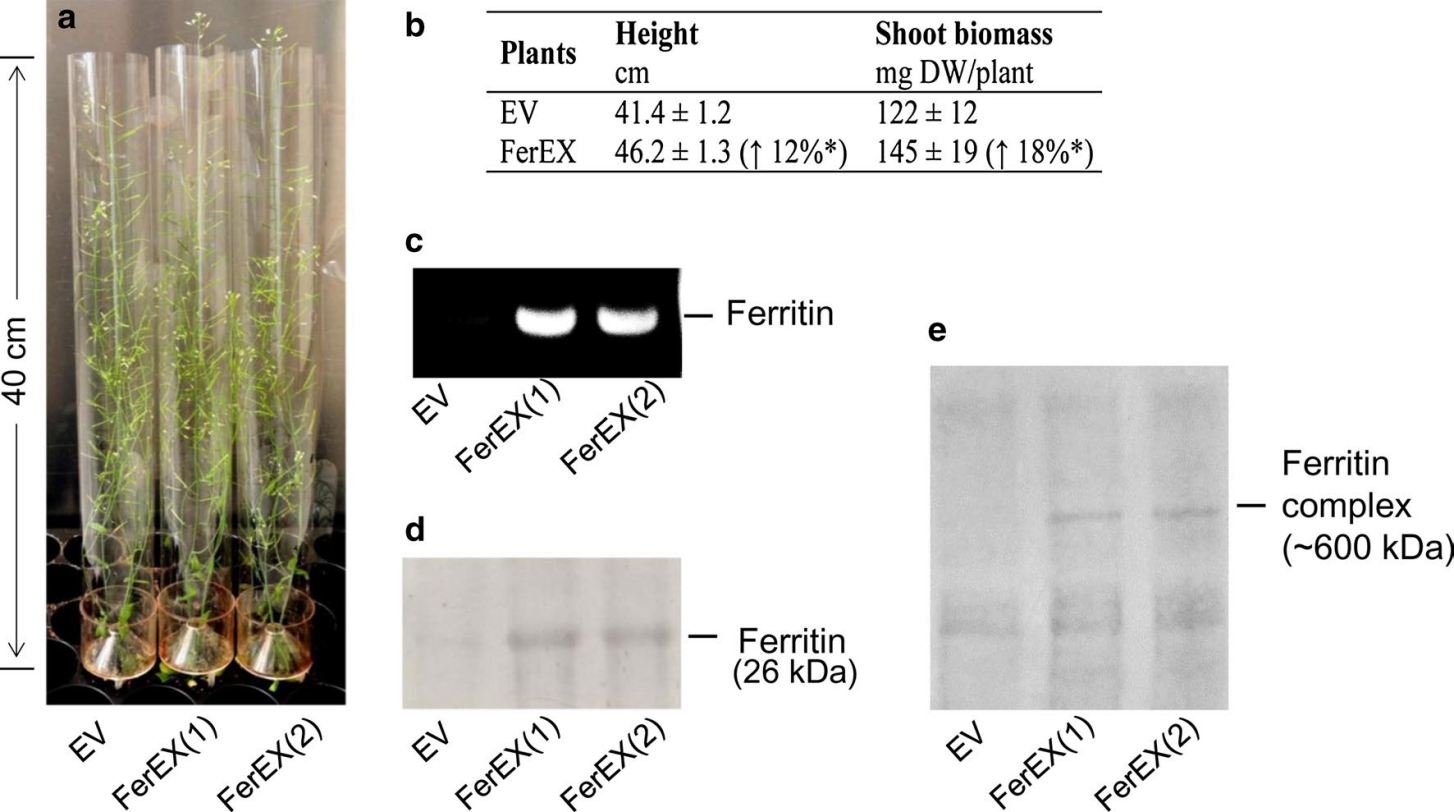
Plant growth images for representative transgenic plants and their Western blot analyses. **a** Iron-fertilized empty vector (EV) transgenic control and the transgenic lines expressing heterologous ferritin secreted extracellularly (FerEX). **b** Plant heights and shoot dry weights (DWs) of the iron-fertilized EV and FerEX plants at the senescent stage. The *percentage values* inside the *brackets* are the increases in height or shoot biomass in FerEX lines compared with the EV control. Values are presented as the mean (±SE) of nine plants for each of the lines. * indicates statistical significance of *p* < 0.05. **c** PCR analysis of genomic DNA extracted from FerEX, confirming the integration of ferritin transgene into the genome of the transgenic *Arabidopsis* lines. **d** Western blotting of ferritin expression levels in cell-wall protein extracts of plant lines after SDS-PAGE, using polyclonal anti-soybean ferritin antibody. **e** Western blotting of ferritin expression levels in cell-wall protein extracts of plant lines after native-PAGE, using polyclonal anti-soybean ferritin antibody. The illustrated transgenic lines: FerEX(1) and (2) represent FerEX-8a and -10g, respectively

To examine the effects of extracellularly expressed ferritin on plant growth, the FerEX transgenic (FerEX-8a and -10g) and empty vector (EV) control plants were grown in parallel under both H_2_O-watering and iron-fertilizing conditions. Under distilled H_2_O-watering conditions, transgenic FerEX plants that overexpressed ferritin extracellularly grew normally as did the EV control plants. Under iron-fertilizing conditions the FerEX transgenic plants showed increased growth compared with the EV control plants, for which the phenotypes of representative plants are shown in Fig. 1a. The average height of FerEX transgenic plants at the senescent stage was 46.2 cm, which was a 12 % (*p* < 0.05) increase over that of the EV control plants. Meanwhile, the average dry weight of FerEX transgenic plant tissues was 145 mg, which was 18 % (*p* < 0.05) increase over that of EV control transgenic plants (Fig. 1b). Other studies have reported increases in biomass yields in transgenic plants of *Arabidopsis* [20], lettuce [25], and tobacco [19] that expressed heterologous ferritin proteins intracellularly. This study, to the best of our knowledge, demonstrated for the first time that similar enhancement phenomenon also exists in *Arabidopsis* expressing heterologous ferritin extracellularly.

**Fig. 2:**
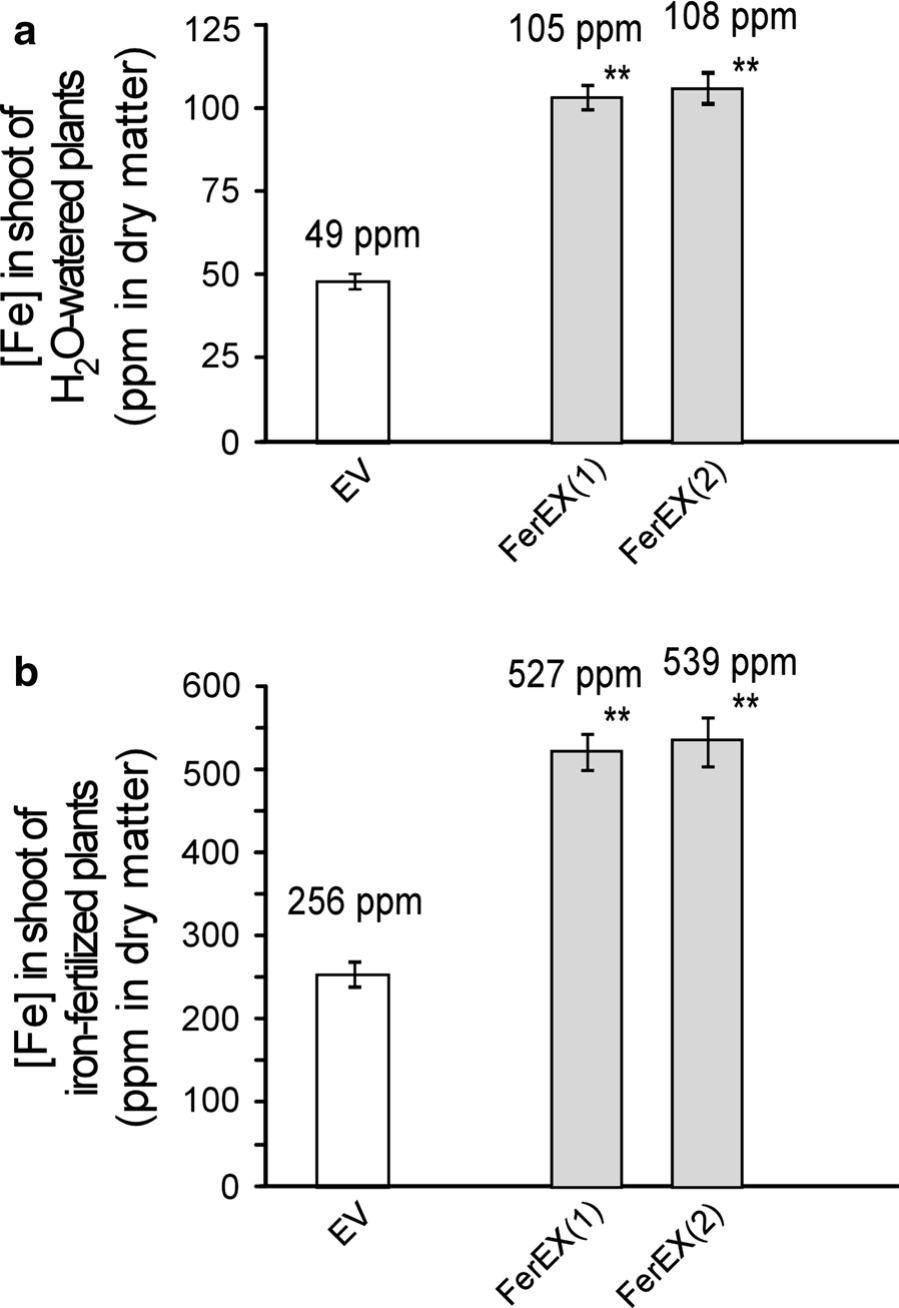
Iron contents in shoot biomass harvested at senescent stage from extracellular ferritin-overexpressing (FerEX) transgenic *Arabidopsis* plants. The plants were H_2_O-watered (**a**) or irrigated with 2 mM Fe-EDDHA (**b**) twice a week during plant growth. Data are presented as the mean (±SE) of five replicates. ** indicates statistical significance of *p* < 0.01 from the empty vector (EV) controls. FerEX(1) and (2) represent FerEX-8a and -10g, respectively

In addition, ICP-OES analysis showed that iron contents in the shoot tissues of FerEX transgenic plants (105–108 ppm in dry matter) was 2.1–2.2 times greater than that of the transgenic EV control plants under normal growth conditions with distilled H_2_O-watering, which suggests that the FerEX plants can be planted without Fe fertilizing but still hyperaccumulate iron from the soil (Fig. 2a). Similarly, under the iron-fertilizing condition, the iron content in the shoot tissues of the FerEX transgenic plants (527–539 ppm in dry matter), was also approximately 2.1 times that of the EV control plants (Fig. 2b). Note that the above iron content levels in FerEX were slightly higher but not statistically significant different from the previously obtained FerIN transgenic Arabidopsis lines that expressed ferritin intracellularly [26]. The latter (FerIN) accumulated iron to a level of 95–100 ppm under normal H_2_O-watering condition, and 514–520 ppm under iron-fertilizing condition [26].

### PCR and Western blot analyses

As described above, initial real-time RT PCR analysis was conducted on the total RNA and converted cDNA for all the independent transformed T1 Arabidopsis FerEx plants, which detected the soybean ferritin transcripts in all these plants. In addition, PCR analysis of the genomic DNA extract from FerEX transgenic lines (FerEX-8a and -10g) confirmed the integration of the heterologous ferritin transgene into the genome of these lines (Fig. 1c).

The expression of the heterologous ferritin protein in FerEX plants was examined by Western blot analysis, using the cell-wall proteins extracted from the shoot tissues collected at the mid-pod stage and the chicken IgY polyclonal antibody against a synthesized soybean ferritin peptide. The Western blot analyses confirm that expression of soybean ferritin in FerEx transgenic plants resulted in a peptide of the expected molecular mass of 26-kDa in denaturing SDS-PAGE (Fig. 1d), as well as an expected molecular mass of about 600-kDa for the ferritin complex in native PAGE (Fig. 1e).

### Iron localization in shoot tissues

**Fig. 3:**
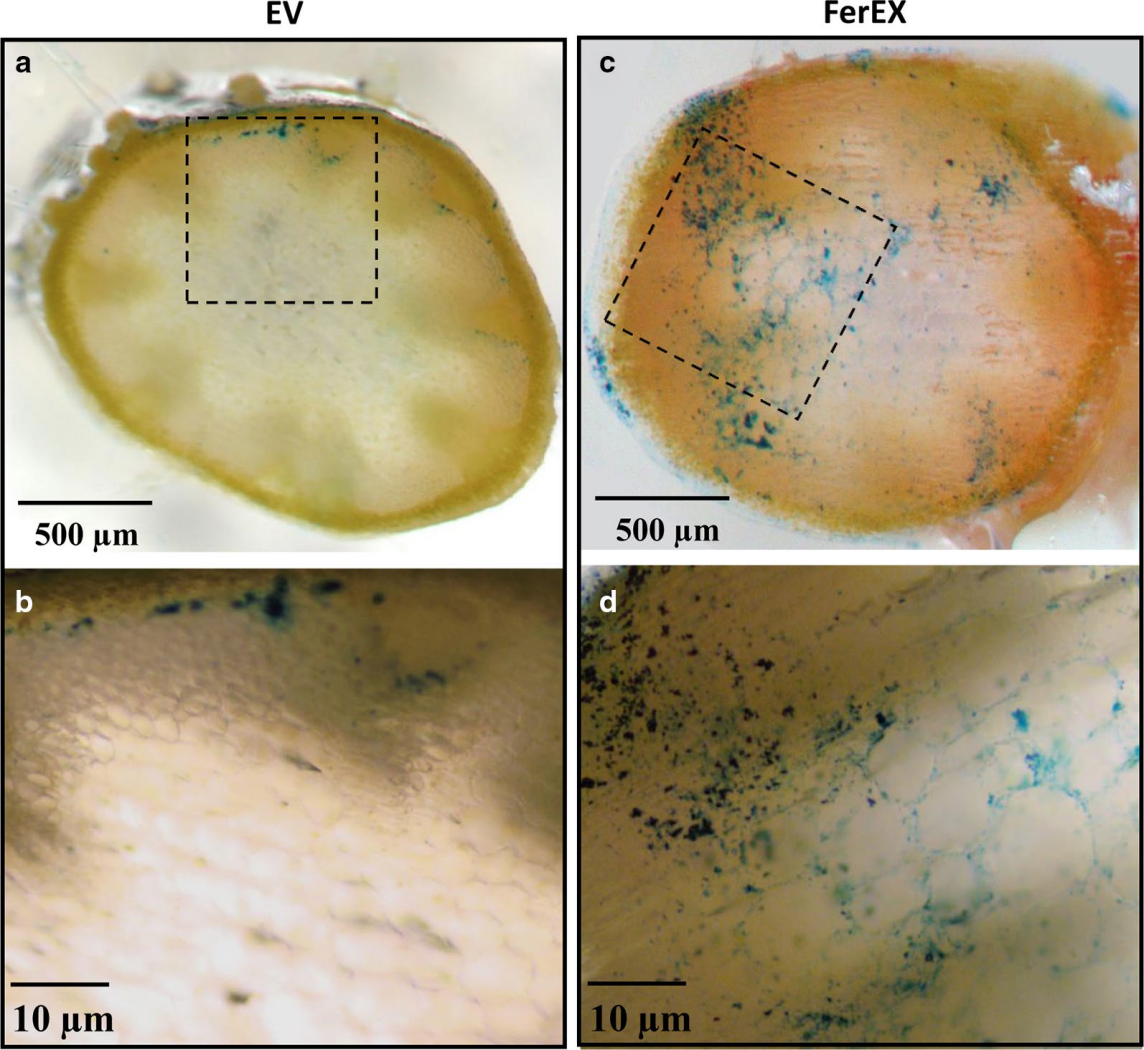
Perls’ Prussian blue staining of cross sections from fresh stem tissues in transgenic *Arabidopsis* plants expressing ferritin that secreted extracellularly. Brightfield optical microscopy showing Perls’ Prussian blue staining of empty vector (EV) control (**a**, **b**), and extracellular ferritin-expressing transgenic plant shoot tissue (FerEX) (**c**, **d**). **b**, **d** are images taken at a higher magnification of the *black boxes* outlined in **a** and **c**, respectively

Optical and X-ray fluorescence microscopy (XFM) were used to investigate iron accumulation at the cellular scale of tissues. First, fresh cross sections of stems from FerEX transgenic and EV control plants were examined by optical microscopy after Perls’ Prussian blue staining [16]. In the EV control plants, some slight blue staining can be detected within the stem sections (Fig. 3a, b). In contrast, significant blue staining signals were detected within the FerEX transgenic plant stem sections, compared to EV control plants, which indicates iron accumulation at higher levels in FerEX transgenic plants (Fig. 3c, d). Under higher magnification, the iron in the EV control plants is deposited at the interfascicular fiber (IFs) between the cortex and the pith parenchyma cells (Fig. 3b), while in the FerEX transgenic plant sections, the deposition of iron is found around the plant cell walls across the whole section (Fig. 3d).

In addition, the images of senesced stems reveal the existence of some faint blue staining in the interior surface of the cell lumen (which is the lumen side of cell walls) in the EV control (Additional file 1: Figure S1A), which is not surprising as endogenous *Arabidopsis* ferritin proteins also exist in the host plant. In contrast, iron deposition was observed in the compound corner middle lamella of FerEX transgenic plants (Additional file 1: Figure S1B, white arrow); such extracellular distribution of iron is consistent with the targeted extracellular region for heterologous ferritin expression in the FerEx transgenic plants.

**Fig. 4:**
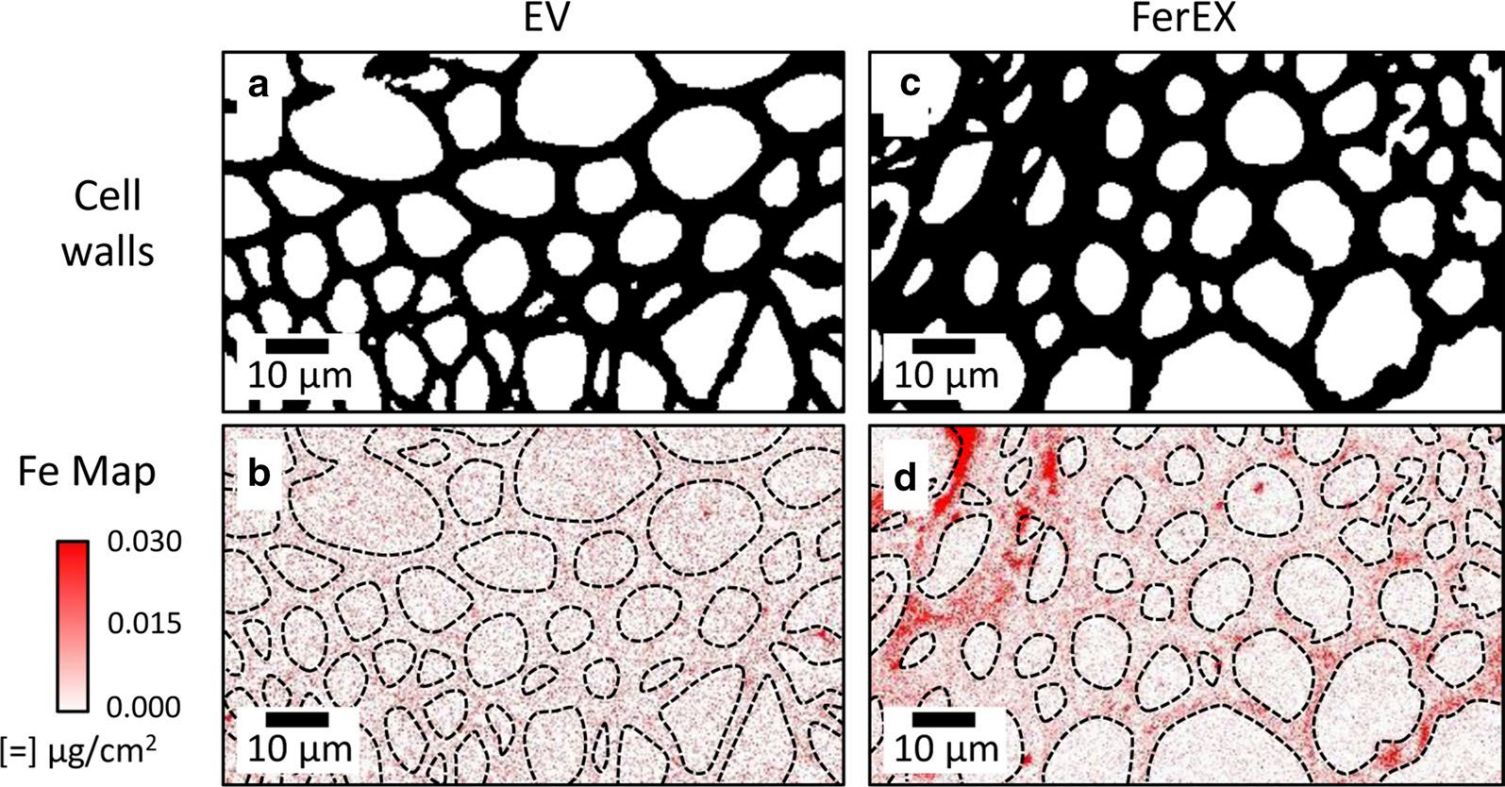
X-ray fluorescence microscopy (XFM) elemental maps for Fe in stem cross sections of extracellular ferritin-expressing and control *Arabidopsis* plants. The 2-micron-thick cross sections were cut from senesced stems from empty vector (EV) control (**a**, **b**) and extracellular ferritin-expressing (FerEX) (**c**, **d**) plants. Cell-wall images (**a**, **c**) were constructed from binary images of potassium XFM maps. The *dashed lines* were drawn from the cell-wall images and overlayed on the iron maps (**b**, **d**) to more easily distinguish iron intensity inside the cell walls. The intensities in both iron maps (**b**, **d**) were scaled the same, and the iron is observed in the FerEx cell walls (**d**) by noting that the iron intensity in the cell walls is higher than the background iron intensity observed in the empty cell lumina

Second, XFM was also used to detect and map iron in 2-micron-thick cross sections cut from EV and FerEX senesced stems (Fig. 4). Results from this highly sensitive elemental mapping technique were in good agreement with those obtained from the Perls’ Prussian blue staining results. XFM maps of iron in the EV stems showed several small, isolated regions of high iron content; however, the iron signal within the cell walls was largely similar to the background over the majority of the image. In contrast, the iron in the FerEX stems (Fig. 4c, d; Additional file 1: Figure S2E–H) could be detected at a level significantly higher than background throughout the cell walls in most regions, but was also observed in elevated concentrations in highly localized areas. The submicron spatial resolution of XFM and the thinness of the sections facilitate the mapping of ions in different cell-wall layers, such as secondary and middle lamella layers [14, 29]. The observation of iron throughout the FerEX cell walls indicates the iron is distributed in both the secondary and compound middle lamella cell-wall layers. This finding suggests that the objective of this study to incorporate iron throughout the entirety wall was generally successful. in addition, similar to the Perls’ Prussian blue staining results, increased iron concentrations could also be detected in some of the corner compound middle lamella regions (Additional file 1: Figure S3, white arrows).

### FerEX transgenic *Arabidopsis*: hot-water pretreatment and co-saccharification

Previously, dilute acid pretreatment was used to assess the pretreatability of FerIN transgenic *Arabidopsis* [26]. In contrast, this study used hot-water pretreatment to evaluate the pretreatability of the biomass, because it is a greener technology that not only benefits the environment, but also avoids the erosion effect of dilute acid to the reactor and eliminates the downstream step of neutralizing the pretreated biomass residue prior to saccharification. In addition, for comparison purposes, the two previously generated intracellular ferritin-overexpressing (FerIN-2a and -4b) transgenic Arabidopsis plants [26] were also grown, harvested, and pretreated side by side with the FerEX plants.

**Fig. 5:**
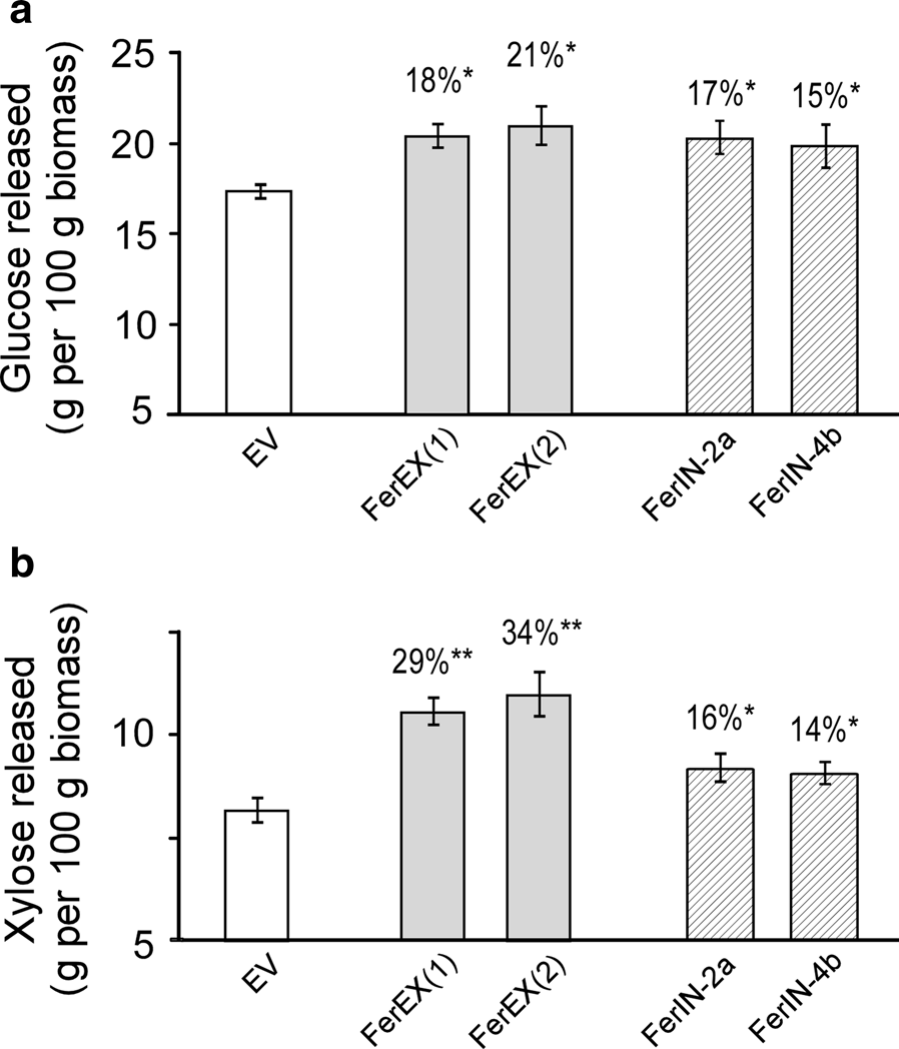
Pretreatability and digestibility of the shoot biomass harvested at senescent stage from extracellular ferritin-overexpressing (FerEX) and control *Arabidopsis* plants. The plants were irrigated with 2 mM Fe-EDDHA twice a week during plant growth. **a** Glucose and **b** xylose release after hot-water pretreatment (180 °C, 17.5 min) and enzyme saccharification with CTec2. EV: empty vector control plant. FerEX(1) and (2) represent transgenic lines FerEX-8a and -10g, respectively. As a comparison, the two previously generated intracellular ferritin-overexpressing (FerIN-2a and -4b) transgenic *Arabidopsis* plants were also grown, harvested, and pretreated side by side with the FerEX plants. Data are presented as the mean (±SE) of five replicates. * indicates statistical significance of *p* < 0.05; ** indicates statistical significance of *p* < 0.01

The dried, ground biomass from iron-fertilized FerEX, FerIN transgenic plants, and the EV control plants was subjected to high-throughput (HTP) hot-water pretreatments at 180 °C, 17.5 min, and after enzymatic saccharification the sugar release was measured. A HTP method that uses 5 mg biomass per well was used because of the small quantity of biomass available from each transgenic plant. The data shows that glucose released after enzymatic hydrolysis from hot-water-pretreated FerEX transgenic plants was similar to FerIN transgenic plants: enhanced 18–21 % more than that found after the hot-water pretreatment and enzymatic hydrolysis of the iron fertilized EV control transgenic plants, which is slightly higher (but not statistically significant) than the extent of enhancement (15–17 %) on glucose release in the FerIN transgenic plants over the EV plants (Fig. 5a).

In contrast, the xylose released from the FerEX transgenic plant biomass was enhanced to a larger extent, i.e., 29–34 % compared eith the EV control plants after hot-water pretreatment and enzyme saccharification, which is significantly higher than the extent of enhancement (14–16 %) on xylose release in FerIN transgenic plants over the EV plant (Fig. 5b). This is a very significant enhancement for the FerEX transgenic plants at these low severity pretreatment conditions. Such observation can be attributed to the facts that FerEX plants accumulate iron in the cell-wall region with a close proximity to cell-wall biopolymers including xylans.

This study showed that FerEX plants enhance more glucose (up to 21 %) and xylose (up to 34 %) release from biomass, which is higher than EV control (Fig. 4); as well as the FerIN plants [26]. Based on the increased shoot dry weight of FerEX plants (18 % more biomass; Fig. 1b) and the sugar release (21 % more glucose and 34 % more xylose; Fig. 5), the sugar yields on a per plant basis is 43 and 58 % more glucose and xylose, respectively, greater than that expected from EV control plants. Theoretically, 100 g of glucose or 100 g of xylose can produce 51.4 g of ethanol [30–32]. Bioethanol production from the improved of sugar release observed in FerEX plants can be calculated following Krishnan et al. [33], in which it was estimated that ethanol yields from glucose and xylose fermentation by recombinant *Saccharomyces* were typically with 95 and 80 % of the theoretical yield, respectively [33]. A more thorough technoeconomic analysis (TEA) can be conducted in the future after transgenic bioenergy plants expressing ferritin extracellularly are grown at large scale.

### Biomass compositional analysis and the implication

It is noteworthy that after hot-water pretreatment and enzymatic digestion of plant biomass, the FerEX plants released 29–34 % more xylose over the EV plant, compared to releasing 18–21 % more glucose over the EV plant (Fig. 5). To address the observed higher extent of xylose release in the FerEX transgenic plants (versus control), we measured the chemical compositions of the harvested EV and FerEX(2) biomasses, using the method described in the "Methods" section. The data revealed that EV biomass contained 33.3 ± 0.6 % cellulose and 12.2 ± 0.4 %, while FerEX(2) biomass contained 33.6 ± 0.3 % cellulose and 12.4 ± 0.3 % (*n* = 3), with no significant differences in their cellulose and xylan compositions between the two lines. It thus appears that the incorporation of heterologous ferritin into the growing wall does not alter wall composition.

Like most other plant proteins, plant ferritins likely have a frequent turnover rate throughout the plant growth phase. The values of the half-lives of ferritin proteins in various species and cells were reported to be in the range of 3.5–72 h [34–36], and it is safe to assume that the value of the half-life of plant ferritin proteins falls within a similar range. We propose that as the expressed extracellular proteins degrade, the released iron ions are initially deposited onto nascent cellulose, hemicelluloses, and lignins, and then quickly diffuse through the continuous polymer matrix of plant cells. Furthermore, because hemicelluloses lack the crystalline structure of cellulose, their availability, and indeed susceptibility to hydrolysis, is greater than cellulose. Eventually, a softened and continuous, interconnecting network that facilitates chemical transport is established in the walls [14, 37].

### *In planta* iron accumulation versus post-harvest supplementation of iron

Although techno-economic analysis for the presented approach of *in planta* iron accumulation in delivering iron catalyst to the plant cell wall has not been conducted, the following two factors support the presented approach of *in planta* iron accumulation may likely be cost efficient and application feasible. First, the localization of iron observed from imaging data supports a new bioprocessing benefit of using a plant-produced ferritin (versus an externally post-harvest exogenously added Fe catalyst), presumably because the accumulated iron ions are in close proximity to cell-wall substrates such as cellulose and hemicellulose in the FerEX transgenic plants. Second, FerEX plants were found to have more shoot biomass than the EV control plants under iron-watering condition (increased by 18 % as indicated in Fig. 1b). Thus, the cost of applying iron fertilization is likely to be offset by the increased plant biomass, as well as the increased pretreatability, digestibility, and the total amount of sugar released.

### Comparison of the intracellularly versus extracellularly approaches

The differences between expressing ferritin in plants intracellularly and extracellularly have two considerations. First, considering that most of the metabolic activities of plant cells take place intracellularly, overexpression of ferritin intracellularly has been demonstrated to provide the plants better protection from oxidative stress, iron toxicity, photoinhibition, and pathogens [21, 23, 24, 38]. The consequences of expressing ferritin extracellularly on plant defenses and other stresses are less clear, and remain to be studied further.

Second, these two approaches definitely showed clear contrast in the pattern for iron deposition in live plants. Our previous study clearly showed that the iron was predominantly deposited on the interior surfaces of cell lumen in FerIN plants [26]; and accordingly, we proposed that at the senescent stage, the intracellularly accumulated iron will be released into the internal (lumenal) surface of cell walls from the broken cells at later growth stages. This release would facilitate the interaction between iron co-catalyst and plant biomass during pretreatment and lead to increased biomass pretreatability.

In contrast, expressing ferritin extracellularly in plants allows the delivery of iron into the plant cell wall, presumably embedded in a sandwich pattern as new cell walls are formed layer by layer. Moreover, this deposition may also create a continuous iron ion diffusion pathway along the hemicellulose network throughout the cell-wall matrix. In this regard, the FerEX approach delivers the iron closer to the plant cell-wall matrix which benefits downstream processing.

Our understanding of the chemical mechanism for the iron enhancement of sugar release during dilute acid and hot-water pretreatments of biomass remains limited. However, from in vitro Fourier transform infrared spectroscopy (FTIR) analysis of treated biomass, we have demonstrated that the *in muro* iron ions during dilute acid pretreatment targets the more recalcitrant chemical bonds in the cell wall, especially the C–O–C and C–H bonds, whereas dilute acid alone targets primarily the glycosyl bonds of polysaccharides [13]. Future studies are warranted to gain deeper understanding of the atomic level interactions between metal ions and cell-wall polymers.

### Efficiency of ferritin secretion using signal peptide and future studies

The expression vector pCAMBIA1305.2 that we used in this study was developed by pCAMBIA (http://www.cambia.org) and contains a rice glycine-rich protein (GRP) signal peptide for extracellular targeting. GRP is one of the major secreted proteins that form the plant cell-wall structure [39]. This vector has been widely used and well demonstrated in literature to express target proteins extracellularly in various plant cells and tissues [40, 41].

Particularly, a recently published study used the above GRP signal peptide in pCAMBIA1305.2 vector and GFP to form pCAMBIA1305.2-SP-GFP, which was named as pSBI-MF11 to transform sugarcane callus cells. The GFP fluorescent microscopic images of the callus cells transformed with pSBI-MF11 showed a clear localization of GFP to the apoplastic space [42]. Such observation effectively demonstrated the efficiency of using GRP signal peptide to direct the secretion of GFP protein to extracellular space of plant cells.

So far, to the best of our knowledge, no heterologous plant ferritins had been reported to be fused to GFP and expressed in transgenic ferritin plants [16–21, 23–25, 38, 43, 44]. The mature ferritin is a 24-mer protein assembly, and it was reported that the *C* termini of many members of the ferritin family tend to be buried during folding [45]. It is unclear if the fusion of a plant ferritin (with a molecular size of approximately 26 kDa for its monomer) to GFP (with a molecular size of approximately 27 kDa) would cause a disruption to the normal self-assembly of ferritin subunits. In addition, the autofluorescence of plant cell wall may also cause interference to microscopic observation of GFP fluorescence. As this complex issue is out of the scope of this study, future studies are warranted to examine the suitability of expressing GFP-ferritin fusion protein in plants for visualizing the localization of target protein.

### Future studies for characterizing ferritin expression and function using model microorganisms

This study has demonstrated a correlation between the iron capture (as shown by Perls’ blue staining and XFM data) and ferritin expression (as shown by the Western blotting analysis of extracted cell-wall proteins) on the plant tissue level. Expressing the soybean ferritin gene, SferH-1, in model microorganisms may be attempted in future work to enable a more precise correlation at the protein level. The expression of bullfrog H-subunit ferritin [46], pea-seed ferritin [47] and soybean seed ferritin [48] have been reported in *E. coli*; in those works, the wild-type ferritins all aggregated as inclusion bodies and were not soluble when heterologously expressed in *E. coli* at 37 °C. Various approaches, including the engineering of the heterologous wild-type ferritins, the lowering of the induction temperature for cell growth, and/or the coexpression of the chaperone molecules had been used to increase the solubility and function of the expressed ferritins in microorganisms [46–48]. Although similar studies are beyond the scope of our current work presented here, future studies to express engineered soybean ferritin in *E. coli* or yeast for confirming the iron capture at the protein level should be conducted.

### Future applications of co-catalyst-accumulating plants for the bioenergy sector

Our previous data demonstrated the effectiveness of expressing ferritin intracellularly in *Arabidopsis* to increase the iron accumulation during plant growth; which enhanced the pretreatability and digestibility of the harvested biomass [26]. Importantly, the extracellularly expressed ferritin *Arabidopsis* plants generated in this study showed a further enhancement the pretreatability and digestibility of the biomass compared with the FerIN transgenic plants (Fig. 5). Together, these studies generating the FerEX plants effectively replace the previous approach of soaking iron containing acid solutions into milled biomass prior to pretreatment, which was time consuming and subject to diffusion limitations. Moreover, this metal co-catalyst accumulation strategy should be “stackable” with other cell-wall engineering approaches, which can lead to a more environment-friendly and economical production of feedstocks for the production of biofuels and biochemicals.

Given the general similarity in cell-wall structure and hemicellulose chemistry, it is feasible that the results shown here for expression of ferritin in *Arabidopsis* plants will extend to poplar (also dicot) and switchgrass (monocot), the representative bioenergy plants.

## Conclusions

This study demonstrates the successful expression of heterologous ferritin protein extracellularly in *Arabidopsis*, which led to significant iron accumulation during the plant growth. Deposition of iron in FerEX cell walls was observed using XFM mapping. in addition, both XFM mapping and Perls’ Prussian blue staining showed a higher iron concentration in the compound middle lamella of FerEX transgenic plants. The harvested transgenic plants showed improved pretreatability and enzymatic digestibility, where significantly more glucose and xylose were released after hot-water pretreatment and enzymatic saccharification. A significantly higher xylose release by that of FerEX biomass points to the additional benefits brought by the extracellular iron accumulation during the plant growth in FerEX plants, compared to the previous intracellular expressing ferritin approach. Future studies will be focused on exploring plant root and rhizosphere microbiome engineering approaches in solubilizing mostly sequestered iron ions in soils, and eventually make the plants independent in the uptake of iron from soils during plant growth.

## Methods

### Plant material and growth conditions

*Arabidopsis thaliana* Columbia-0 (Col-0) was used as the wild-type (WT) parent line for the transformation with soybean ferritin gene. The germination of WT seeds on ½ MS agar medium, the transfer of seedlings to soil (Metro-Mix 360, SunGro Horticulture, Canada) in pots, the placing of plant pots under light shelves (ArabiSun Lighting System, Lehle Seeds, Texas, USA), and the plant growth conditions were described previously [26].

### Codon optimization and gene synthesis

**Fig. 6:**
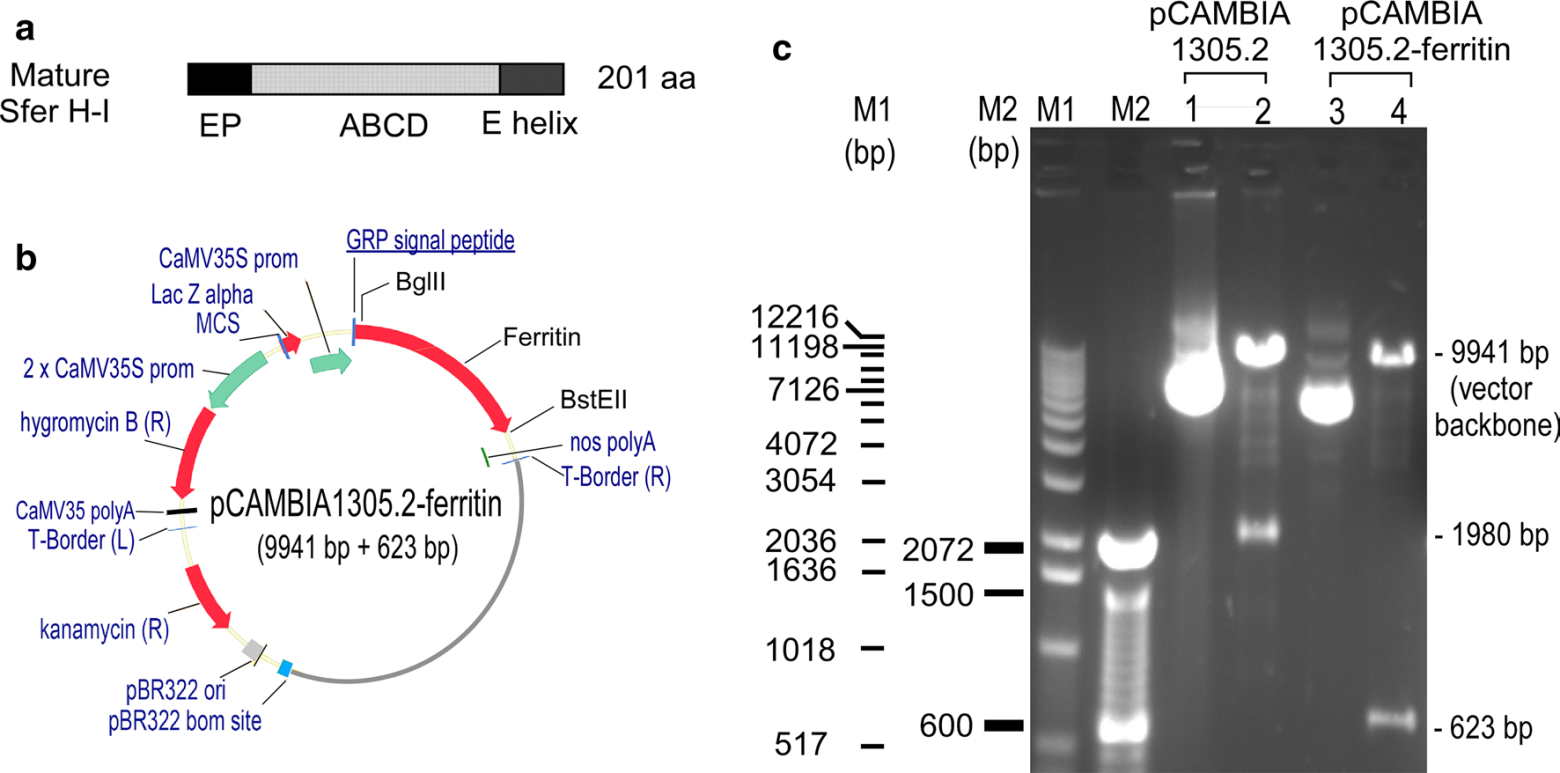
Construct for expressing soybean ferritin extracellularly in *Arabidopsis*. **a** Mature form of soybean ferritin H-1 (Sfer H-I) was used for gene synthesis. EP: the sequence encoding extension peptide; ABCD and E, the sequences corresponding to the A to E helixes of mature ferritin. **b** Vector pCAMBIA1305.2 was developed by pCAMBIA (http://www.cambia.org), which has a glycine-rich protein (GRP) signal peptide for extracellular targeting. For expression construct, the catalase intron-GusPlus gene cassette in above vector was replaced by the ferritin gene; nopaline synthase (nos) polyA was the terminator. **c** Confirmation of construct pCAMBIA1305.2-ferritin by restriction enzyme digestion. *Lanes 1* and *2*, uncut and BglII-BstEII cut empty vector (1305.2), respectively; the latter was digested into two bands, including the 1980 bp GUSplus gene. *Lanes 3* and *4*, uncut and BglII-BstEII cut construct (pCAMBIA1305.2-ferritin), respectively; the latter was digested into two bands, including the 623 bp ferritin gene

The diagram of transit peptide (TP)-deleted, mature form of soybean ferritin (SferH-1, with a GenBank accession no. of M64337) is illustrated in Fig. 6a. As described in the literature, the TP is needed only for delivering ferritin precursor to plastids [43], thus it was removed in this study for expressing ferritin extracellularly in *Arabidopsis*. The nucleotide sequence for SferH-1 mature protein was codon-optimized according to the codon-usage frequencies of *Arabidopsis*, and synthesized with a BglII restriction cut site at the 5′-end and a BstEII cut site at the 3′-end by GenScript (Piscataway, NJ). The synthesized gene was cloned into the *E. coli* vector pUC57 and amplified.

### Gene constructs

The synthesized ferritin gene cloned in PUC57 were cut with BglII-BstEII, and ligated to BglII-BstEII cut binary expression vector pCAMBIA 1305.2 (GenBank accession no. AF354046), which has a signal peptide for secreting the expressed protein into the apoplastic spaces in *Arabidopsis* tissues. The vector also contains the hygromycin B resistance gene (hpt II) as the plant selection marker, for selection of transformed *Arabidopsis* plants. The expression of the inserted codon-usage-optimized ferritin gene is driven by the CaMV35S promoter (Fig. 6b, c).

### *Agrobacterium*-mediated transformation

The above construct, as well as the empty vector pCAMBIA 1305.2 was introduced into competent *Agrobacterium tumefaciens* cells (strain C58) using a freeze–thaw method [49]. Positive colonies were restreaked onto fresh LB plates containing rifampicin (10 µg/mL), kanamycin (50 µg/mL), and checked by PCR for the presence of the heterologous ferritin gene, using the forward primer (ACCCACGAGGAGCATCGTGG) and reverse primer (GGTCACCTTATCAATCTAAC). The construct-carrying *A. tumefaciens* strain C58 was used to transform WT *Arabidopsis* Col-0, using an *Agrobacterium*-mediated floral dip transformation procedure [50]. The transformed plants were grown to senescent stage to collect the seeds, which are a mixture of nontransformed and primary transformed seeds (T1). These T1 seeds were continued to the next step for the selection of homozygous transformants.

### Selection of homozygous transgenic plants

In the following steps, ½ MS agar medium containing 1 % sucrose supplemented with 20 mg/L hygromycin B is referred to as MS-hygromycin medium, and the definition of T1, T2 and T3 seeds is in accordance with the literature [51]. T1 seeds were germinated on the MS-hygromycin medium, and the hygromycin-resistant seedlings were then grown in soil and self-pollinated to generate T2 seeds representing individual transformation events. For selecting homozygous plants, T2 seeds were germinated on MS-hygromycin medium. The numbers of hygromycin-resistant and sensitive seedlings for each transformation event were used for a Chi squared test to select plants with a single transgene insertion (with a typical 3:1 segregation ratio, for hygromycin-resistant versus -sensitive). Ten T2 seedlings from each single-insertion event were further grown in soil (one seedling per pot). T3 seeds were harvested from individual plants and germinated using the MS-hygromycin medium. The homozygous plants were confirmed only if T3 seeds could germinate to produce 100 % hygromycin-resistant plants without segregation, and these plants were used for genomic DNA and total RNA extraction followed by genomic integration and transcript analyses.

### Genomic DNA isolation and genomic integration confirmation

The aforementioned segregation-test-screened T3 homozygous lines were used to confirm genomic integration. Genomic DNA was isolated from 2 to 3 *Arabidopsis* young leafs (~100 mg wet weight; approximately 3 cm^2^ in total area) using DNeasy 96 Plant Mini Kit (Qiagen, Valencia, California), and used to detect the presence of ferritin transgene in the genome of transgenic plants by PCR using specific primers (as described above) targeting the synthesized soybean ferritin gene.

### Total RNA extraction and transgene transcript detection

The confirmed homozygous transgenic lines from each gene construct’s transformation were used to confirm the expression of ferritin genes. Total RNA was extracted from 15 to 30 mg (fresh weight) liquid nitrogen ground young leaves of transgenic and empty vector control *Arabidopsis* plants with 1 mL TRIzol reagent (Invitrogen, Carlsbad, CA). The extracted total RNAs were treated with DNase I (Invitrogen) to remove genomic DNA contamination. One microgram of purified total RNA was used for reverse transcription using SuperScript III Reverse Transcriptase with random primers (Invitrogen) based on the manufacturer’s kit manual. The obtained cDNA samples were stored at −20 °C until used for the real-time RT (reverse transcription) PCR amplification using the forward primer (GAATACAACGCTTCATATGTGTACCA) and reverse primer (AGCGTGCTCCCTTTCCTCTT) for targeting the synthesized soybean ferritin gene and using the forward primer (GGCTCCTCTTAACCCAAAGGC) and reverse primer (CACACCATCACCAGAATCCAGC) for targeting *Arabidopsis* actin 2 (At3g18780). Real time RT-PCR was conducted as previously described [52].

### Plant iron accumulation determination

Transgenic plants were grown with iron fertilization to test their iron accumulation capacity. Fe-ethylenediaminedi(*o*-hydroxyphenylacetic) acid (Fe-EDDHA) in the concentration of 2 mM was used as the iron fertilizer to irrigate *Arabidopsis* plants as previously reported in the literature [23]. To test the upper limit of concentration for iron fertilizer that *Arabidopsis* plants can tolerate, we also tested the spraying of plants twice a week with 20 mM Fe-EDDHA, and found that this concentration inhibited plant growth of *Arabidopsis*. Thus, 2 mM Fe-EDDHA was used through the studies for plants irrigated with iron fertilizer twice a week. The pots with plants of empty vector (EV) control and transgenic seedlings were placed on a board, on which the location of pots was randomized, in a greenhouse with 16-h light (200–300 µmol/m^2^ s^1^), 8-h dark cycles at 24 °C. Plants were harvested at the mid-pod stage, and rinsed with ddH_2_O for three times by centrifugation so that no residual iron on the surface of plant tissues would affect the measurement of biomass iron content.

Dry shoot samples were ground and passed through a 20-mesh (1 mm) screen using a Wiley Mill, and an aliquot was used to measure the metal concentrations using a procedure modified from the literature [20, 53, 54]. In brief, 20 mg of dry ground sample was digested with 0.4 mL 25 % (v/v) nitric acid (Trace Metal Grade, Fisher Scientific), at 70 °C, overnight. Following digestion, the extracts were diluted to 5 mL with fresh Millipore (Synergy water Purification System) deionized H_2_O (the final nitric acid concentration was 2 %), and the concentrations of Fe and other metal ions were measured using inductively coupled plasma—optical emission spectroscopy (ICP-OES) by the Chemical Analysis Laboratory at the University of Georgia. Trial experiments established that the recovery rates of the metal ions during the nitric acid-extraction process were between 97 and 102 %, and thus the original data are being presented without normalization adjustments.

### Cell-wall isolation

Mid-pod stage plant shoots were used for cell-wall isolation, using the protocol described by Seara et al. [55] with modification according to Watson et al. [56]. Five grams of *Arabidopsis* shoot tissues of WT or FerEX were collected, ground in liquid nitrogen, and homogenized in ice-cold lysis buffer (0.12 M Tris–HCl (pH 7.4), 50 mM NaCl, 30 mM sodium ascorbate, 5 % glycerol, 5 % (w/w) polyvinylpolypyrrolidone (PVPP) and protease inhibitor (cOmplete, EDTA-free Protease Inhibitor Cocktail; Roche, Basel, Switzerland). The homogenate was filtered through Miracloth (catalog no. 475855; EMD Millipore, Billerica, Massachusetts) and washed by 250 mL of ice-cold washing buffer (10 mM Na acetate, pH 5.5) before cell-wall protein extraction.

### Cell-wall protein extraction

Cell-wall protein was extracted as described in Jiménez et al. [57]. The freshly isolated cell wall was incubated with 10 mM Na-citrate/phosphate (pH 5.5) supplemented with 1 M NaCl at 4 °C for 48 h. The cell-wall suspension was filtered through Miracloth, and the protein extract was dialyzed against 20 mM Na-acetate buffer (pH 5.0) with three changes of the buffer. The dialyzed protein extract was centrifuged at 6500×*g* for 25 min and concentrated using Amicon Ultra-15 Centrifugal Filter Unit with Ultracel-3 membrane (catalog no. UFC900308, EMD Millipore, Billerica, Massachusetts). The concentration of proteins was estimated according to the method of Bradford Protein Assay Kit (catalog no. 5000002; Bio-Rad, Baltimore, Maryland, USA).

### Antibody against soybean ferritin peptide and Western blot analysis after SDS-PAGE and native PAGE

**Fig. 7:**
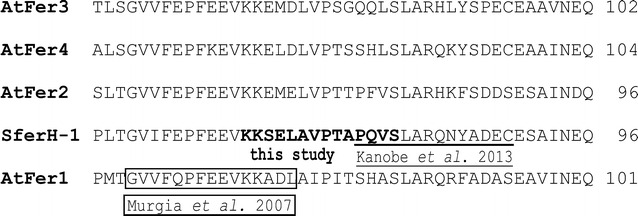
Amino acid alignment of soybean ferritin SferH-1 with *Arabidopsis* ferritins and selection of specific peptide that can distinguish SferH-1 from the endogenous host *Arabidopsis* ferritins. The first 49 amino acids of SferH-1 (GenBank accession no. M64337) is the transit peptide (TP) for delivery to plastids and is excised in the mature protein, thus it is excluded from the alignment. *Arabidopsis* has four ferritin genes that include from AtFER1 to AtFER4 (At5g01600, At3g11050, At3g56090, and At2g40300, respectively). The selected peptide KKSELAVPTAPQVS is predicted to be antigenic and highlighted in *boldface*. The *underlined* peptide is the segment that was used by Kanobe et al. [44], for preparing anti-soybean ferritin antibody. The *boxed* peptide sequence was picked by Murgia et al. [58], for *Arabidopsis* ferritin antibody preparation

The first generation of anti-soybean ferritin antibodies reported in the literature was prepared by using extracted soybean ferritin as antigen to inoculate the animals [18, 20]. As more ferritin gene amino acid sequences became available, a second generation of ferritin antibodies were made using synthesized soybean ferritin peptides [44]. In this study, the amino acid sequence of soybean ferritin SferH-1 was aligned with the endogenous host *Arabidopsis* ferritins, and a peptide was selected ensuring that it can be immunologically distinguished from host Arabidopsis endogenous ferritins (Fig. 7). It is noteworthy that the picked peptide sequence is adjoining to those peptides used to make anti-*Arabidopsis* ferritin [58] or anti-soybean ferritin [44] antibodies, suggesting some consistency between independent research groups in choosing adjoining amino acid regions that are predicted to be antigenic. In our work, the selected soybean ferritin peptide was synthesized and used to prepare purified chicken IgY polyclonal antibodies using the Custom Antibody Services of Pierce Biotechnology (Thermo Scientific, Rockford, IL, USA). The chicks and chickens were fed exclusively feed without soybean products or meal for their entire lives to avoid cross reactivity with other soybean ferritin epitopes than the epitope of the specific peptide selected from soybean ferritin expressed in these transgenic plants.

For SDS-PAGE, 20 µg of cell-wall proteins extracted from shoot tissues were mixed with 4× Laemmli sample buffer in a 3:1 v/v mixture, and were separated on Invitrogen NuPAGE Novex 4–12 % Bis–Tris Mini Gels; for native-PAGE, 30 µg of cell-wall proteins from shoot tissues were processed using NativePAGE Sample Prep Kit (ThermoFisher, Waltham, Massachusetts) following manufacturer’s protocol and subjected to the separation, using the Invitrogen NativePAGE 4–12 % Bis–Tris Gels. After the gels were resolved they were transferred to polyvinylidene difluoride (PVDF) membrane, using Invitrogen iBlot Gel Transfer System. Western blots were carried out using a SNAP-ID Western blotting system (Millipore, Billerica, MA). The above prepared chicken IgY polyclonal antibody against soybean ferritin was used as the first antibody with a dilution of 1:500 in SuperBlock T20 (PBS) blocking buffer (ThermoFisher, Waltham, Massachusetts) for 10-min incubation, followed by washing three times with PBS/Tween-20. The second antibody was goat polyclonal secondary antibody to chicken IgY—H&L, which was conjugated with alkaline phosphatase (catalogue no. ab97142, Abcam, Cambridge, MA) and used a dilution of 1:2000 in SuperBlock T20 (PBS) blocking buffer for 10-min incubation followed by washing. Alkaline Phosphatase activity was visualized by adding the NBT/BCIP chromogenic substrates (Invitrogen) in water after submerging the PVDF membranes.

### Perls’ Prussian blue staining and confocal scanning laser microscopy for subcellular distribution of Fe accumulation

Perls’ Prussian blue staining was conducted using a procedure modified from the literature for the localization of iron in plant tissues [16]; the procedure in details was described previously [26]. In brief, samples of fresh or senesced stems (approximately 0.5-cm long segments, cut from stem 1 cm above the soil surface) were used for the staining, using the fleshly prepared 2 % potassium ferrocyanide and 2 % hydrochloric acid as the staining buffer. Brightfield images were acquired using a SPOT RTKE CCD camera (Diagnostic Instruments, Sterling Heights, MI), which was equipped on a Nikon E800 microscope (Nikon, Tokyo, Japan) platform.

### X-ray fluorescence microscopy (XFM)

Two-micrometer-thick cross sections were prepared using a diamond knife fit into a Leica EM UC7 Ultramicrotome (Wetzlar, Germany) from dried EV and FerEX transgenic Arabidopsis plant stems. The sections were mounted to the surface of a Norcada 200 nm thick silicon nitride window (Edmonton, AB, Canada) by placing sections into small droplets of water placed on the window surface. The sections were found to be adhered to the window after the water evaporated. XFM was performed at beamline 2-ID-E at the Advanced Photon Source at Argonne National Laboratory (Argonne, IL, USA). The incident X-ray beam energy was 10.2 keV and spot size was approximately 0.5 µm in diameter. Elemental maps were built in 0.3-µm steps with 5-ms dwell times at each step. XFM data analysis was performed using MAPS software [59]. In brief, the full spectra were fitted to modified Gaussian peaks, the background was iteratively calculated and subtracted, and the results were compared to standard reference materials (NBS 1832 and 1833, NIST).

### Biomass compositional analysis

Compositional analysis of the harvested plant biomass was performed by using method described in literature [60].

### Hot-water pretreatment and enzymatic digestion of plant biomass

The procedure was similar to that described in our recent publication [61]. In brief, transgenic plants were harvested, air-dried, ground, and passed through a 20-mesh (1 mm) screen by using a Wiley Mill (Model 3383-L10; Thomas Scientific, Swedesboro, NJ, USA), and then tested for total sugar release using a high-throughput (HTP) method that combines hot-water pretreatment with enzymatic hydrolysis [62]. The HTP-type pretreatment procedure allows the processing of large numbers of biomass samples with small sample amounts to measure sugar release [62–65]. In brief, ground biomasses of 5 mg of from empty vector transgenic *Arabidopsis* control plants and FeEX transgenic *Arabidopsis* plants were weighed in sample replicates of 5 mg into random individual wells on 96-well Hastelloy plates; ddH_2_O was added, the plates sealed with Teflon tape, clamped, and subjected to hot-water pretreatment at 180 °C for 17.5 min, followed by enzymatic saccharification. Enzymatic saccharification was carried out by adding buffer to each well in the pretreated plate, mixing, and using Novozymes CTec2 at loadings of 70 mg enzyme/g biomass with incubation carried out at 40 °C for 70 h. After enzymatic saccharification, the sugar release was measured using a glucose oxidase/peroxidase (GOPOD) assay for glucose and a xylose dehydrogenase (XDH) assay for xylose absorbances versus standard curves as described previously [62].

## Additional file

**Additional file 1.** Additional figures S1 to S3.

## Abbreviations

35S: enhanced cauliflower mosaic virus 35S promoter
ddH_2_O: distilled deionized water
EV: empty vector control transgenic plants
FerEX: extracellularly expressed ferritin transgenic plants
FerIN: intracellularly expressed ferritin transgenic plants
HPLC: high-performance liquid chromatography
hpt: hygromycin phosphotransferase encoding gene
HTP: high throughput
nosT: nopaline synthase terminator
XFM: X-ray fluorescence microscopy
