# Supplementary material for: Directed plant cell-wall accumulation of iron: embedding co-catalyst for efficient biomass conversion
Source: Biotechnol Biofuels. 2016 Oct 21;9:225. doi: 10.1186/s13068-016-0639-2 (PMC5073452; doi:10.1186/s13068-016-0639-2)
Supplement: Supplementary file 1 — Additional file 1. Additional figures S1 to S3. [file 13068_2016_639_MOESM1_ESM.pdf]

Manuscript title:

Directed plant cell wall accumulation of iron: Embedding co-catalyst for efficient biomass conversion

Supplemental figures.

File type: PDF

Contents:

Supplemental Figure S1. Fe detection in in the senesced stem tissues of transgenic *Arabidopsis* plants using the Perls' Prussian blue staining.

Supplemental Figure S2. X-ray fluorescence microscopy (XFM) maps of Fe in cross-sections of stems in extracellular ferritin-expressing and control plants.

Supplemental Figure S3. Potassium and iron colocation map showing higher accumulations of iron in some of the FerEX corner compound middle lamellae.

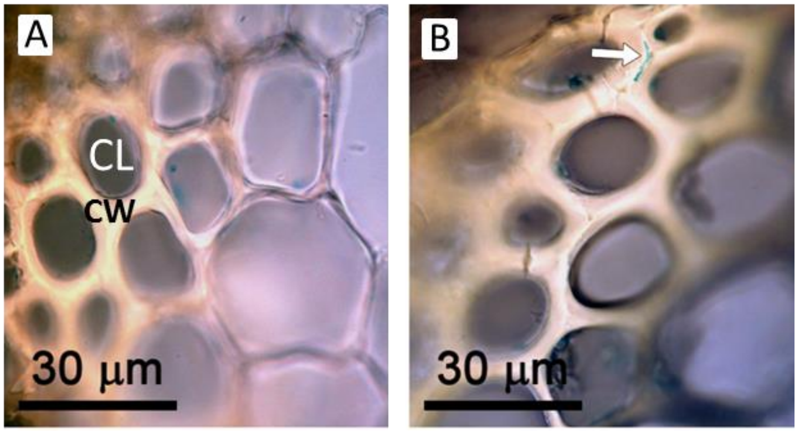

Supplemental Figure S1. Fe detection in in the senesced stem tissues of transgenic *Arabidopsis* plants using the Perls' Prussian blue staining. Brightfield optical microscopy showing Perls' Prussian blue staining of empty vector (EV) control (**A**), and extracellular ferritin-expressing transgenic plant shoot tissue (FerEX) (**B**). White arrow in (**B**) indicates Fe localized within the compound middle lamella. CL, cell lumen; CW, cell wall.

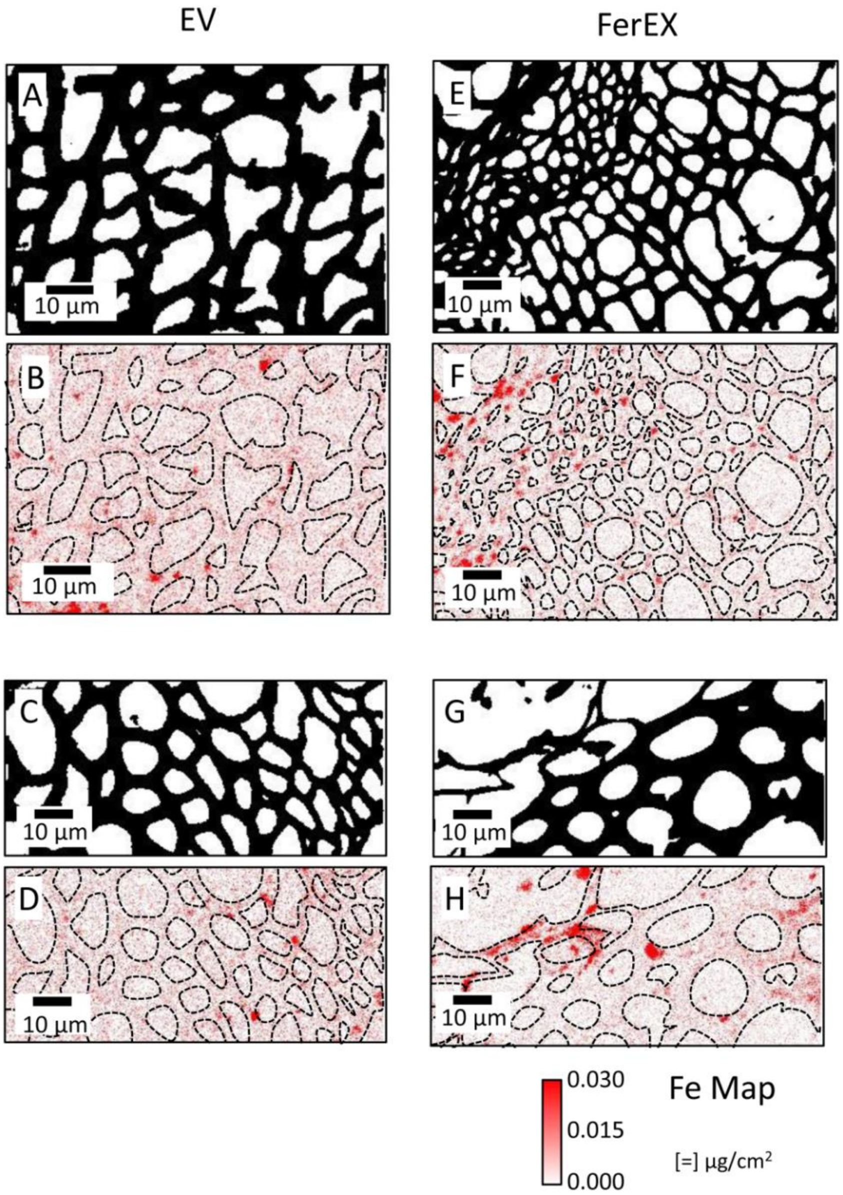

Supplemental Figure S2. X-ray fluorescence microscopy (XFM) maps of Fe in cross-sections of stems in extracellular ferritin-expressing and control plants. The 2-micron-thick cross-sections were cut from senesced stems from empty vector (EV) control (**A**-**D**) and extracellular ferritin-expressing (FerEX) (**E-H**) *Arabidopsis* plants. Cell wall images (**A**, **C, E, and G**) were constructed from binary images of potassium XFM maps. The dashed lines were drawn from the cell wall images and overlayed on the iron maps (**B**, **D, F, H**) to more easily distinguish iron intensity inside the cell walls. The intensities in all iron maps (**B**, **D, F, H**) were scaled the same and the iron can be observed in the FerEx cell walls (**F, H**) by noting that the iron intensity in the cell walls is higher than the background iron intensity observed in the empty cell lumina.

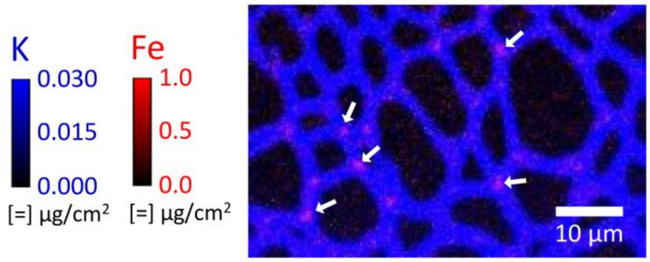

Supplemental Figure S3. Potassium and iron colocation map showing higher accumulations of iron in some of the FerEX corner compound middle lamellae (white arrows). The potassium and iron ion maps were obtained from 2-micron-thick cross-sections cut from FerEX senesced stems using X-ray fluorescence microscopy (XFM).
